# Book Reviews

**Published:** 1903-02

**Authors:** 


### BOOK REVIEWS.

How to Succeed in the Practice of Medicine. By Joseph McDowell Mathews, M. D., LL. D., President Kentucky State Board of Health; Professor of Surgery, Hospital Medical College, Louisville; Author of "Mathews on Diseases of the Rectum." Octavo, pp. 234. Illustrated. Louisville: John P. Morton & Company. 1902. [Price, $2.00.]

The distinguished author of this useful book has been a teacher and practitioner for more than twenty-five years, hence is in a position to know of what kind of advice students and recent graduates stand in greatest need. Those who know Professor Mathews best would expect him to write just such a book as this which, from cover to cover, abounds in good nature—human nature we might say with great propriety—and is a heart to heart talk between teacher and pupil. Moreover, it contains so much of value to every physician and it is put into such a readable shape that it is not likely to reach alone the office table of the junior, but to be found in the hands of every physician who would be entertained and instructed in the general success of the members of his profession. Besides, it is a book for the doctor's family and this will add to its effectiveness as well as to its popularity.

In the amphitheater and in the lecture room, Professor Mathews is noted for his clearness of description, simplicity of rhetoric, and forcefulness of diction. He is also a public speaker of wide-spread fame, in which capacity he makes potent use of the same qualifications that have given him such strength as a teacher. From all this it might be expected that a book from his pen on such a subject as that under consideration would be entertaining.

How to succeed in the practice of medicine! Who among the juniors would not like to know the secret? How many of the middle-aged would not wish to turn backward to the open sesame? And who of the seniors in medicine would not wish they could have secured entrance to the charmed portals at the outset?

But there is no assured road to success in any occupation, much less is there one to professional success in medicine. In order to succeed one must be prepared in the most careful way. Unpreparedness is the bane of the period. To be well prepared for a difficult work, is to rob its approach of half its perplexities. But preparation means time and this in turn, means money; hence many, too many, enter the ranks of medicine with scant preparation for the trials that await them.

This book does not tell one how to succeed in spite of preparation, but it points out the road to success after proper equipment for the journey. It gives the plain rules that experience teaches to be a safe guide, and it imparts that kind of knowledge which can be gained only by experience and attentive observation. It also tells how to avoid errors too often committed, which is a matter of supreme importance, especially in the beginning of the professional journey.

In the course of its pages it takes the young practitioner through the long and trying ordeal of beginning and waiting, up to success and fame, and finally, to the close of his career, when in the evening of life he enjoys the fruits of his labor. The story is entertaining from beginning to end and bespeaks the work of a master. It is in reality one of the best recent books on a subject not strictly scientific, but of closely allied importance to the technical studies of the physician.

A SYSTEM OF PHYSIOLOGICAL THERAPEUTICS. A Practical Exposition of the methods, other than Drug-giving, useful in the prevention of Disease and in the Treatment of the Sick. Edited by SOLOMON SOLIS COHEN, A.M., M.D., Senior Assistant Professor of Clinical Medicine in Jefferson Medical College; Physician to the Jefferson Medical College Hospital, and to the Philadelphia, Jewish and Rush Hospitals. In 11 volumes. Volume V. Prophylaxis, Personal Hygiene, Civic Hygiene, Care of the Sick. By Joseph McFarland, M. D., Professor of Pathology, Medico-Chirurgical College, Philadelphia; Henry Leffman, M. D., Professor of Chemistry in the Woman's Medical College, Philadelphia; Albert Abrams, A. M., M. D., formerly Professor of Pathology, Cooper Medical College, San Francisco; and W. Wayne Babcock, M. D., Lecturer on Pathology and Bacteriology, Medico-Chirurgical College, Philadelphia. Octavo, pp. 856. Illustrated Philadelphia: P. Blakiston's Son & Co. 1903. [Price for the set, $27.50 net.]

This volume deals with the natural history of medicine and gives important facts as far as at present understood concerning the origin, dissemination, and prevention of disease. It is in reality an introduction to the science of medicine, but it is also much more, since its special province is to set forth in plain terms, instructions as to the preservation of health and to teach how to prevent disease. These in the present day constitute the highest province of medicine and the most exalted functions of the physician.

In the first part the origin and prevention of diseases are discussed by McFarland and Babcock; in the second part civic hygiene is dealt with by Henry Leffman; and in the third part domestic and personal hygiene, nursing and care of the sick-room are delineated by Albert Abrams. These parts are subdivided into sections and these again into chapters, so easy reference may be had to any topic or branch of the general subject. It is doubtful if so much information of equal value can be obtained elsewhere for the price. Moreover, it is just the kind of information with which every physician should become familiar. The entire series is of great moment, but this book is indispensable for every medical practitioner.

Practical Midwifery. A Manual of Obstetrics for Students and Physicians By Edward Reynolds, M. D., formerly Instructor in Obstetrics, and Franklin S. Newell, M. D., Assistant in Obstetrics and Gynecology in Harvard University Medical School, Boston. In one 8vo volume of 531 pages, with 253 engravings and 3 full-page colored plates. Philadelphia and New York: Lea Brothers & Co. 1902. [Cloth, $3.75 net.]

The authors of this work admit the congested condition of medical literature, but justify another book by themselves because an earlier volume received favor and, further, because the publishers think there is demand for a book constructed on the lines drawn for this manual.

Admitting all this to be true we still feel that both authors and publishers should be extremely circumspect in offering new books to an already overtaxed profession. The literature of obstetrics has been made rich of late by the publication of comprehensive volumes with a wealth of illustration most remarkable. Nevertheless, we see in this book some advantages, or perhaps excellent features, that commend it to favorable reception. It is a compact book, concise in its statements, betrays experience in teaching, and represents the advanced scientific thought of the present day. In support of the latter statement, or indeed, of all in the foregoing paragraph, we cite what the authors say under the head of abortion, miscarriage, and premature labor. There is a constant misapplication of these terms and it would serve to clear the way to complete understanding and intelligence on these topics, if teachers would adopt in concert the classification here made—namely, abortions are those terminations of pregnancy that occur during the first three months, or until the placenta becomes formed; miscarriages, from the end of the third until the end of the seventh month, or from the formation of the placenta until the viability of the fetus; and premature labor after the fetus has become viable.

In the section upon abnormal labor the authors are strong in the presentation of their views and we commend this part of the book as worthy of special study by junior practitioners. There has been throughout the work a judicious selection of illustrations, and though not profuse they are clear in demonstrating the text and are well executed for the most part.

It is a treatise likely to commend itself to the thoughtful student and ambitious obstetrician, into whose hands it will surely fall.

---

A Reference Handbook of the Medical Sciences. Embracing the Entire Range of Scientific and Practical Medicine and Allied Science. By various writers. A new edition, completely revised and rewritten. Edited by Albert H. Buck, M. D., New York City. Volume V. Illustrated by chromolithographs and 576 half-tone and wood engravings. Nine volumes, imperial 8vo. New York: William Wood & Co. 1902. [Price, muslin, $6.00 per volume; leather, $7.00 per volume; half morocco, $8.00 per vol.]

There are one hundred and forty-eight contributors to this volume, of whom four reside in Buffalo. Their names are Drs. Montgomery A. Crockett, Herbert M. Hill, Roswell Park and Grover W. Wende. Their work compares favorably with that of other contributors to the book, which in many respects is a notable one, maintaining the high standard set by the preceding volumes. While we cannot take up each article in detail, we may point out some of the excellencies which await the subscriber to this series as he examines the fifth volume.

In the first place there is an extensive presentation of the subject of inflammation, which is always one of interest and has never been exhausted. Then follows a symposium on insanity contributed by some of the prominent alienists of this country, which is well illustrated and makes a treatise on the subject of 125 double column quarto pages. Kidney and liver diseases are similarly treated, as well as affections of the larynx and lungs. The diseases of the eye are scattered through the entire series of eight volumes, this one containing its full share. The group of articles relating to the upper-air tract, when associated with the symposium on diseases of the ear in volume four, and the article on diseases of the nasal cavity to appear in volume six, constitute one of the most complete expositions on diseases of the ear, nose and throat that has been printed in English.

Besides these groups there are isolated articles of great importance, written by prominent teachers and authors, as well as laboratory workers, that add to the value of the volume and thus increases the scientific worth of the series. These are only a few of the excellent features of volume five, that appear as one turns its pages rather cursorily, but a critical examination discloses so much treasure that we are unable to catalogue it without trying the patience of the reader.

It is sufficient to say, as a concluding thought, that this volume is full of valuable information for general practitioner and specialist,—a work well arranged, beautifully illustrated and splendidly printed.

---

A MANUAL OF SURGERY. For Students and Practitioners. By WILLIAM ROSE, M. B., B. S., Lond., F. R. C. S., Professor of Clinical Surgery in King's College, London, and Senior Surgeon to King's College Hospital, and ALBERT CARLESS, M. S., Lond., F. R. C. S., Surgeon to King's College Hospital and Teacher of Operative Surgery in King's College, London; Examiner in Surgery to Glasgow University. Fifth edition. Octavo, pp. 1227. Illustrated. New York: William Wood & Co. 1902. [Price, $5.00 net.]

A new edition of this popular manual will be welcomed by a great number of practitioners as well as students, though it was originally designed especially to meet the needs of the latter, the large textbook being too much to master during undergraduate career. Be this as it may, there is place for the book which is attested by the fact that four editions already have been exhausted, the last one having been issued only a year before the fifth or present edition.

The authors have made some changes of value in this issue, though we question whether the time honored traditions relating to inflammation should be cast aside to give first place to bacteriology and the principles of antiseptic and aseptic surgery. However much we may value the work of the laboratory as a clinical aid, and we would not belittle it, the fact, nevertheless, remains that the antemortem pathology learned at the bedside is the most practical and the laboratory contributions to this end are invaluable. One must master the classics of inflammation before the laboratory aids can be appreciated or applied.

This work is a compact, scientific, and intelligent exposition of modern surgery and commends itself to everyone interested in the study or practice of that branch of medicine.

Manual of Gynecology. By Henry T. Byford, M. D., Professor of Gynecology and Clinical Gynecology in the College of Physicians and Surgeons, Chicago; Professor of Gynecology in the Post-Graduate Medical School and in the Chicago Clinical School. Third revised edition. Duodecimo, pp. 620. Illustrated. Philadelphia: P. Blakiston's Son & Co. 1902. [Price, $3.00 net.]

We have made favorable comment of this book heretofore in these columns, and it is a genuine pleasure to take up the subject again with this new edition as a text. The original intention of the author was to supply the student with a work that would meet the requirements of undergraduate study better than the more pretentious textbooks; also to furnish the practitioner who made no pretentions to specialism, a guide that would enable him to decide when he has reached his limitations and when the gynecologist was needed. It must be a satisfaction to Professor Byford to have succeeded so admirably in both directions.

There has been a rearrangement of the parts in this edition, so that now there is consecutive progress from anatomy, through surgery to functional and nervous diseases, making a homogeneous treatise that appeals to the natural sense of order that should pervade the medical mind.

The marginal notes are serviceable devices that every student will appreciate. The illustrations have been chosen with care and are artistic delineations of the text, adding to the usefulness and mechanical beauty of the book. In all the domain of gynecological literature there is no minor treatise that surpasses it for scientific accuracy, and concise description.

---

PROGRESSIVE MEDICINE. Volume IV., December, 1902. A Quarterly Digest of Advances, Discoveries and Improvements in the Medical and Surgical Sciences. Edited by HOBART AMORY HARE, M. D., Professor of Therapeutics and Materia Medica in the Jefferson Medical College of Philadelphia. Octavo, cloth, 412 pages, 54 illustrations. Philadelphia and New York: Lea Brothers & Co. [Per volume, $2.50, by express prepaid; per annum, in four cloth-bound volumes, $10.00.]

This volume closes the series for 1902, which in its four books has been replete with valuable information not found elsewhere in such accessible form. This book deals with diseases of the digestive tract and allied organs—the liver, pancreas and peritoneum; anesthetics, fractures, dislocations, amputations, surgery of the extremities, and orthopedics; genitourinary diseases; diseases of the kidneys; physiology; hygiene, and closes with a practical therapeutic referendum. The contributors of these articles in the order named are Max Einhorn, Joseph C. Bloodgood, William T. Belfield, John Rose Bradford, Albert P. Brubaker, Charles Harrington, and E. Q. Thornton.

The surgical article is of special interest, particularly those portions dealing with anesthetics, fractures and operative technique. Where there is such a wealth of excellent material, it is, however, difficult to determine which part is of most value, there being in these instances quite as much pertaining to general medicine and hygiene that deserves careful examination as the surgical topics. The therapeutic referendum is the best compilation for the year that we have seen.

KIRKE'S HANDBOOK OF PHYSIOLOGY. Revised by WILLIAM H. ROCKWELL, JR., M. D., and CHARLES L. DANA, A. M., M. D., Professor of Diseases of the Nervous System, Cornell University Medical College, New York; Physician to Bellevue Hospital. Octavo, pp. 865. Seventeenth American edition. With upwards of 500 illustrations, many in colors. New York: William Wood & Co. 1902. [Price, $3.00 net.]

This sterling handbook, which has appeared in repeatedly revised editions for so many years, has contributed to the knowledge of physiology in no small degree. Its arrangement is such as to make it a favorite with the undergraduate, while the practitioner prizes it as a work of reference. The present edition has undergone such necessary revision as will serve to keep it well in the front rank, physiological chemistry having received special attention. Again, that portion dealing with the blood has received merited consideration, while illustrations have been changed in some instances to suit the demands of progress.

It is a physiological handbook that has stood the test of time and is to be commended for its scientific usefulness, as well as for its freshened appearance.

---

THE MEDICAL DIRECTORY OF NEW YORK, NEW JERSEY AND CONNECTICUT. Published by the New York State Medical Association, 64 Madison Avenue, New York. Volume IV. 1902-1903.

This useful directory is again presented in an attractive form. It contains 13,364 names, 10,606, of which belong to the State of New York; 1,655 to New Jersey, and 1,103 to Connecticut. The use of tinted paper to designate the different sections of the book is a good plan because it facilitates reference. The difficulties in making a complete directory are many, and physicians themselves are to blame for defects in these important books of reference. This one appears to be as nearly perfect as it is possible to make such a work.

A Pocket Textbook of Materia Medica, Therapeutics, Prescription Writing, Medical Latin and Medical Pharmacy. By William Schleif, Ph. G., M. D , Instructor in Pharmacy in the University of Pennsylvania. New (2d) edition, revised and enlarged. In one 12mo volume of 382 pages. Lea's Series of Pocket Textbooks. Edited by Bern B. Gallaudet, M. D. 1902. [Cloth, $1.75 net; limp leather, $2 25 net.]

Materia medica and its allied topics furnish material for voluminous textbooks and subjects for studious attention. It is surprising how much of such a very large branch of medical science has been put into this condensed form, and that, too, without doing violence to the general purpose of the main subject or its associated topics. It is an excellent book for the student from which to refresh his thought, and for the practitioner as a ready reference handbook.

This edition, though so soon called for, has been completely overhauled and the revision furnishes new text, as well as a revamping of the old, so that the book represents the immediate present in its teaching. Among the changes may be mentioned an enlarged presentation of medical Latin and prescription-writing, and a new section entitled an annotated therapeutic index of new remedies.

